# An approach for assessing risk, planning, and decision-making in protecting ecological resources on contaminated sites compared to local regions

**DOI:** 10.1007/s10661-025-13993-9

**Published:** 2025-04-29

**Authors:** Joanna Burger, Michael Gochfeld, Kevin G. Brown, Kelly Ng, David S. Kosson

**Affiliations:** 1 Division of Life Sciences, Rutgers University, 604 Allison Road, Piscataway, NJ 08854 USA; 2 Environmental and Occupational Health Sciences Institute (EOHSI), Rutgers University, Piscataway, NJ 08854 USA; 3 Consortium for Risk Evaluation with Stakeholder Participation (CRESP), Vanderbilt University, Nashville, USA; 4 Consortium for Risk Evaluation with Stakeholder Participation (CRESP), Rutgers University, Piscataway, USA; 5 Rutgers Robert Wood Johnson Medical School, EOHSI and CRESP, Piscataway, NJ 08854 USA; 6 CRESP and Civil and Environmental Engineering, Vanderbilt University, Nashville, TN 37215 USA

**Keywords:** Ecological risk, Land use, Department of Energy, Risk communication, Environmental metrics, Temperate forest

## Abstract

The United States and other countries have degraded lands because of legacy wastes from the Second World War, Cold War, and industrialization. There is a need to return lands to productive uses that necessitates assessing and monitoring ecological resources. Federal governments and the public are interested in assurances that federal landowners are protective of environmental and ecological health. This paper uses the U.S. National Land Cover Database to (1) compare land cover on two Department of Energy (DOE) facilities with the surrounding region (10-km and 30-km bands), (2) determine if each has preserved more of the climax vegetation on each site, and (3) discuss how the method allows managers, regulators, and the public to assess if ecological resources on contaminated lands are protected. The analysis method employed provides a monitoring tool that can be used following restoration or management. About 70% of Oak Ridge Reservation (ORR) is forested, compared to 45% of the 10-km buffer (52% of 30-km buffer). Savannah River Site (SRS) protected 58% of its forest compared to 27% of its buffers. Both DOE sites have the opportunity to preserve the largest tracts of unbroken forest during remediation, especially those that include wetlands surrounded by forest. The highest percentage of land cover on both sites is the local natural forest. Visually, ORR has more development in the surrounding region than does SRS. This method can be applied to degraded sites across the U.S. and elsewhere and provides a visual tool for managers, regulators, and the public to quickly access information on vegetation types, the importance of ecological resources, and vulnerability of these resources within the region.

## Introduction

Legacy wastes from the Second World War, the Cold War, and subsequent industrial development have left the United States (U.S.), European Nations, and other countries with contaminated lands. Many U.S. Federal and Tribal governmental agencies, state governments, and the public want to transform such land to productive uses (DOE 1994a, b, 1999; Hou and Tabbaa, 2014; Brunner & Rechberger, 2015). Some Department of Energy (DOE) sites are slated to release land to other state or federal agencies, while some will remain in DOE control. Potential uses for such lands include protecting existing natural habitats, community development, agriculture, residences, and recreational opportunities. These pressures require both assessment tools and monitoring programs to ensure continued protection of ecological lands.

Further, there is interest in providing and maintaining green spaces for human health (Galvani et al., 2016). Recent research has demonstrated that “green spaces” within or near communities contribute to human health benefits, independently of aesthetic values (Akpinar et al., 2016; Kingsley, 2019). These include providing indirect and direct benefits on human mental health, cardiovascular health, and reduction of mortality (van den Berg et al., 2015; Sinnett et al., 2022; CDC 2022). Thus, decreasing amounts of green spaces on degraded lands that are adjacent to neighbors and communities might pose a risk not only to natural ecosystems themselves, but to human health and well-being. In contrast, ensuring continuity of green space with adjacent communities could provide valuable community health benefits.

Protecting the environment on degraded lands, and during remediation and restoration, includes not only providing clean water and air, but also intact ecosystems and the eco-cultural services the ecosystems provide (EPA, 2004, 2024a; Davidson, 2013; Costanza et al., 2017). Protection of existing wild lands and reclamation of contaminated and otherwise degraded lands is an important societal goal (Lamb et al. 2019; Galvani et al., 2016; Costanza et al., 2017). Further, there are competing future use visions and claims for how restored lands should be used in the future, and who should benefit (Bohnee et al., 2011; Burger, 2022a, 2022b; Chan et al., 2012).

The land on degraded sites can be reclaimed by remediation, by active restoration, by allowing the contaminated or damaged land to undergo succession to the regional vegetation community, or by a combination of these (Cairns, 1994). In Europe and many other countries, the term for reclaiming contaminated land, particularly from nuclear facilities, is called “decommissioning and environmental remediation” (NEA 2023), but the process is the same. The aim of environmental remediation is to remove the risk of harm to human health and the environment from buildings and facilities, equipment, and activity sites no longer in use that pose significant challenges for countries with nuclear waste (Chatzis, 2016; Gil-Cerezo et al., 2017).

Before determining the desired future condition of degraded lands, we suggest that the on-site value of ecological resources should be determined, as well as the importance of those resources in the region. Such characterization will provide science-based guidance to help prioritize different potential uses based on risk and relative values. One technique is to select bioindicators as surrogates for the many species, unique habitats, and interests (Parrott, 2010; Siddig et al. 2016). One key characteristic of ecosystems is the predominant vegetation type, often called the climax community. The prevailing climax vegetation in a region usually includes most of the typical plant and animal species within that ecosystem. Climax refers to the vegetation that would normally occur given the local climate, topography, soil, and geology, given sufficient time (sometimes called mature vegetation, [Forman, 1995]) and absence of intensive human activity (e.g., agriculture, grazing, logging). The prevailing view has been that succession is a developmental process whereby the final stage of vegetation (= climax vegetation) is determined by regional climate and other factors (Meeker & Merkel, 1984). Others have argued that a variety of environmental factors could produce different types of climax vegetation (van der Valk, 2014). For the purposes of this paper, we refer to the predominant, mature, native vegetation community for the region as the climax community.

Understanding local land use cover types in relation to the local climax vegetation type will provide key information for future monitoring plans. Characterization will also provide useful information to a range of stakeholders who are affected and interested in protecting intact ecosystems and reducing the risks to the climax vegetation type. Characterizing ecological resources is a difficult task because of the thousands of species, populations, interactions, and physical environments of ecosystems. The difficulty is determining how to conduct such a characterization, determine what might be at risk, determine what eco-cultural values are important to local communities, and develop a toolkit of methods that can be applied regionally and nationally (Burger, 2006, 2019). Additionally, ecological characterizations are essential to understanding other factors, such as sustainability (Hou and Al-Tabbaa 2014; Hou et al., 2014; Cappuyns, 2016; Cvitanovi et al. 2016), equity (Bullard, 1990; EPA, 2009, 2019), and existence values (Davidson, 2013) in such characterizations. That is, for some people, it is important to know that certain ecological resources exist, even if they never expect to see or use them (e.g., polar bears in the Arctic).

In this paper, we propose a method (e.g., a screening tool) to examine one indicator of ecological health within a site (e.g., amount of climax vegetation) and test its applicability to compare sites to the surrounding landscape and region, and to compare between sites. We use two of the major DOE sites as case studies because there are many former industrial sites across the country that need evaluation and prioritization (for action and funding). DOE has one of the largest cleanup tasks in the world, and prioritization is an important component of scheduling activities (Crowley & Ahearne, 2002, DOE, 2022a). The two sites are Oak Ridge Reservation (ORR) in Tennessee and Savannah River Site (SRS) in South Carolina. We used these two sites because they are in the same ecoregion: Eastern Temperate Forest (EPA, 2024a). The indicator we chose was land cover (land use), which can be examined using the National Land Cover Database (NLCD, 2019) and that provides coverage of the entire U.S. Land cover includes information on human development, agriculture, and ecological classes of vegetation cover (among others). The NLCD can be used to compare land-cover type abundance at multiple scales, providing managers, regulators, and the public with an indication of whether a site has more of a given vegetation ecotype than the surrounding regions, and whether there are connections across the boundaries.

The methods applied here provide a broadly applicable tool for assessing whether landowners (in this case DOE) are protecting as much or more of the local climax vegetation on-site as occurs off-site. The method can be applied across many different types of degraded and contaminated sites, in many places. The method allows comparison of resource protection on any site, regardless of the dominant climax community of the region. This can form the basis to address the risk to the climax vegetation from remediation or the physical expansion of other on-site activities. One limitation of our analysis, which reflects a snapshot in time, is that it cannot assess the risk or protective value of proposed changes in use to current climax vegetation without additional information.

Our specific objectives were to (1) assess the relative representation of different land use types on each site, (2) compare land use on each site to the surrounding landscape, (3) compare the relative representation of the climax vegetation (in this case, eastern temperate forest), (4) compare the amount and type of development on each site, and (5) demonstrate the use of the climax vegetation as a bioindicator of ecological protection that can be applied to assessment and monitoring. We selected 10-km and 30-km bands (buffers) because the former encompasses the potential risk and loss of green spaces to neighbors and local communities, and the latter encompasses a greater region that could include other protected lands such as parks, wildlife management areas, and forests (Burger et al., 2023). Ecological information on all three parcels (site, 10-km buffer, and 30-km buffer) provides information useful to stakeholders for evaluating whether DOE or other landowners are protective of the environment (PCCRAM 1997; EPA, 2009; Chakraborty et al., 2016; Goodman & Thompson, 2017; McCauley & Heffron, 2018; DOE 2020; Burger et al., 2022a, 2022b).

In addition, we wanted to see whether climax vegetation (often a surrogate for the health of locally common and rare species) could be used to compare protectiveness on different contaminated sites, even when climax vegetation differs. Although both ORR and SRS are in the same temperate forest ecoregion, one natural ecosystem is deciduous (ORR), and the other is coniferous (SRS) (EPA, 2024a). The application of the method to different ecoregions will be useful for managers, regulators, and the public to have a consistent method of comparing some aspects of protectiveness across regions that range from forests to deserts. This is the first step in that process.

## Background on department of energy

DOE-Environmental Management (DOE-EM) has 16 degraded sites that will require 50 years or so to complete remediation (Government Accounting Office) at a cost of about $377 billion (NRC 2019; DOE, 2017). Protecting human health and the environment is one of DOE’s cleanup goals (DOE, 2022a). Some previously remediated DOE lands have been released for other land uses, but more will be released after remediation. Because decisions on the final land use and disposition for DOE legacy sites may be delayed for decades, it is important to develop transparent and consistent tools to assess the risks to both humans and the environment (NRC 2019). There is a reciprocal relationship between cleanup goals and future land use, as well as the extent of cleanups allowing residential use of some sites, industrial use of others, and perhaps no use at some (e.g., protected natural ecosystems) (Gochfeld et al., 2015).

On the larger DOE sites, only about 10% of the land was industrialized; the rest was allowed to undergo natural succession. In the absence of development and other human intrusions, these areas are some of the most pristine, undisturbed land in the U.S. (Brown, 1998; Burger et al., 2019; DOE, 1994a; Parr et al., 2003; Whicker et al., 2004). This protection contrasts with rapid development in some of the surrounding areas and communities. Most DOE sites developed land use plans that described future use of the sites once cleanup is completed (DOE, 1980; Gochfeld et al., 2015). Even with such future use plans, it is important to assure regulators and the public that ecological resources on site are still protected.

## Methods

### Overall protocol

Our overall approach was to (1) examine land use/land cover on each site using the National Land Cover Database (NLCD, 2019), (2) select the key indicator at each (temperate forest), (3) compare temperate forest on each with the surrounding 10-km and 30-km bands (buffers) using the NLCD, and (3) compare the two sites with respect to their protection of the natural climax vegetation (EPA, 2024a). On each site, the climax vegetation is temperate forest, although ORR is primarily deciduous, and SRS is primarily coniferous.

### Study sites

**Fig. 1:**
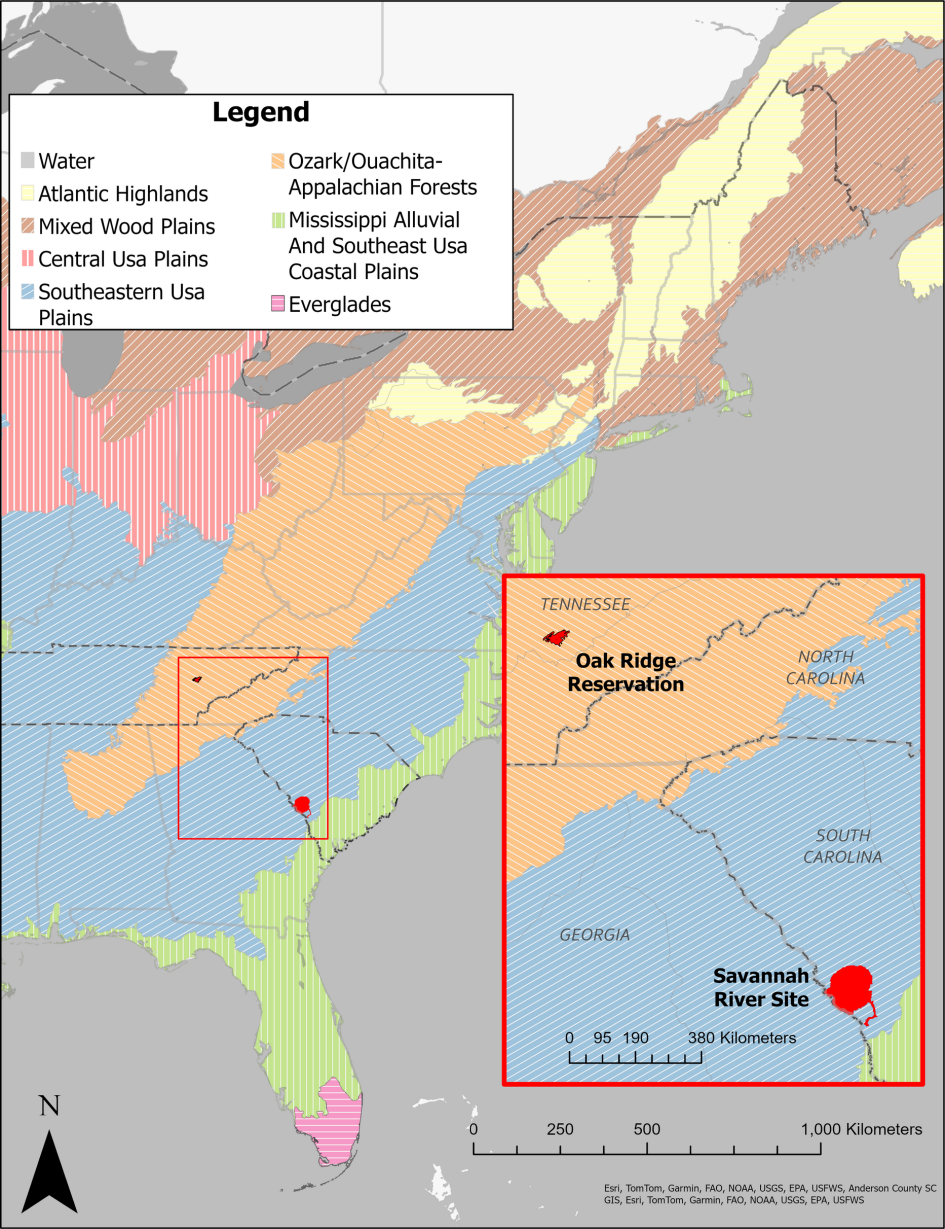
Map showing locations of Oak Ridge Reservation and Savannah River Site in the eastern United States with their ecoregions. Both sites are in the Level 1 Eastern Temperate Forest. Oak Ridge Reservation is in the Ozark/Ouachita-Appalachian forests. The Savannah River Site is in the southeastern U.S. Plains (EPA, 2024a)

Oak Ridge Reservation (ORR) is in the valley and ridge landscape of East Tennessee (Fig. 1). It has an area of land of approximately 13,400 ha and is bordered on the north and east by the City of Oak Ridge and on the south and west by the Clinch River and Melton Hill Lake impoundment (Energy Communities Alliance (ECA), 2020). The hill and valley topography results in an array of different ecological conditions (Parr and Hughes 2006; Giffen et al. 2009, 2011, 2012a, b). When DOE moved farmers off the land in the 1940 s (where it had been extensively logged), DOE used about 10% of the land for their facilities, leaving the rest as a security buffer. In the latter areas, ecological succession following cessation of agricultural activities resulted in this land returning to deciduous forest (Johnston 2012; Parr et al., 2003). Oak Ridge was mainly involved with uranium enrichment (Department of Energy (DOE), 1980, 2022b).

The Savannah River Site (SRS), located in South Carolina, is bordered on the southwest by the Savannah River and encompasses about 80,178 ha (Fig. 1). In the early 1950 s, the people and businesses of six towns were relocated to allow construction of the SRS plant devoted to the production of plutonium and tritium for national defense (DOE, 2022b). Again, only about 10% of the land was devoted for operations, and the rest served as a buffer protected by security. While under DOE management, buffer lands reverted to coniferous forest.

Both sites have a current mission of contributing to DOE’s overall energy, defense, nuclear non-proliferation, and research activities (DOE, 2022b, c). Cleanup from the Cold War and other activities are conducted by DOE-EM (Environmental Management). One of their mandates is safe cleanup while protecting human health and the environment. Continuing activities on site requires maintenance and construction of facilities to serve that mission.

### Selection of key indicator

This paper assesses the relative abundance of land use types within DOE facilities and compares abundance to that within 10- and 30-km buffers around each. The 10-km band around the site was selected because it includes neighborhoods, towns, or cities that are adjacent to the site and might suffer harm or benefit from biological resources or activities on the site. The 30-km band was chosen to capture more of the regional ecology. Although these buffer distance selections are arbitrary, they allow comparison of ecological resources on many different sized facilities across the U.S. (Burger et al., 2023). Moreover, the objective is to show proof of concept, and individual communities can select different sized buffers for comparison to fit their economic and cultural needs. Global Information Systems (GIS) and other forms of land cover analysis have proven useful in comparing different sites and habitat types (Antunes et al., 2001; Applestein & Germino, 2021, 2022). The U.S. Geological Survey has distributed the multi-resolution NCLD at about every 5-year intervals since 1992 (www.mrlc.gov/data/ncld-land-cover-conus-all-years). The NLCD also has been used to examine land use changes over time (Homer et al., 2020).

### Analysis of current land cover/land use

The NLCD (2019) database has numerous ecological land use types that can be combined, depending upon objectives. That is, there are different types of forest and different levels of development (low to very dense; NLCD, 2016). For ORR and SRS, there were 15 land cover types identified. Our initial analysis combined forest types (deciduous, coniferous, and mixed) and development types (open space, low intensity, medium intensity, and high intensity) for comparison to other NLCD habitats (Table 1). Secondary analysis examined the abundance of the three forest types and the relative abundance of different development cover types (NLCD, 2019).

**Table 1:** Comparison of land cover type percentages on Oak Ridge Reservation and Savannah River Site with the surrounding buffers. Given are percentages for each land cover. Forest includes all NLDC forest types, and developed includes all NLDC development types

Land cover type	DOE site	10-km buffer band	30-km buffer band
**Oak Ridge Reservation**			
Total ha	13,400	90,609	453,216
Open water	4	4	4
Developed	16	30	22
Forest	70	45	52
Shrub, grassland	2	2	2
Woody, herb wetland	2	< 1	< 1
Agriculture	6	18	18
Barren land	< 1	< 1	< 1
**Savannah River Site**			
Total ha	80,178	175,934	677,100
Open water	2	< 1	< 1
Developed	4	6	10
Forest	58*	28	28
Shrub, grassland	10	20	16
Woody, herb wetland	25	25	25
Agriculture	< 1	20	20
Barren land	< 1	< 1	< 1

To determine differences among the three areas (site, 10-km band area, 30-km band area), we used analysis of variance (ANOVA). To perform the ANOVA, each area (site, 10-km area, and 30-km area) was divided into equal-area polygons, each of which was assigned a cover type. Although complete polygons were of equal sizes, some polygons on the border were smaller, and their area contributed a relative percentage to the overall totals of each cover type. The number of polygons for the site, 10-km buffer and 30-km buffer, was as follows: (1) ORR 40, 218, and 890 and (2) SRS 16,376, 79,062, and 279,274.

To provide quality assessment maps and analyses, we used Google Earth satellite data to examine areas that seemed to be unusual as a function of the surrounding habitat (i.e., a small block of open area surrounded by forest with no roads or obvious human connections). To check which groups differed significantly, a post hoc Tukey test was used to test the difference between each site and the 10-km buffer and between the site and the 30-km buffer. The data were analyzed using Python (version Python 3.8 and Spyder Anaconda). We also used the chi-square test for differences among types of development and forests (Statistical Analytical Systems (SAS), 2020). We considered *P* < 0.05 as significant.

## Results

### Land use on oak ridge reservation and Savannah river site

**Fig. 2:**
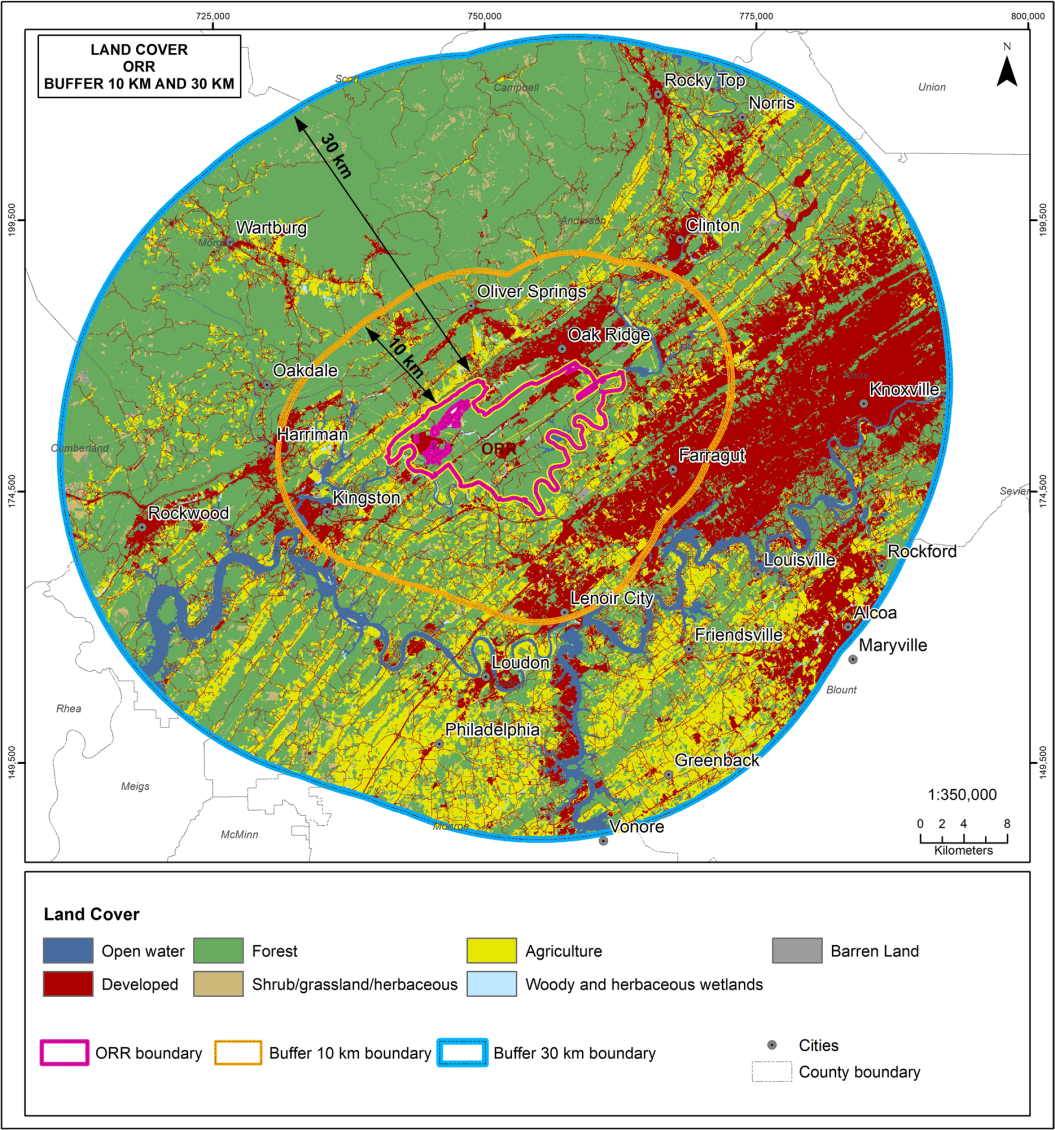
Land cover on Oak Ridge Reservation (boundary in red) and the 10-km and 30-km buffer. Most of the development off-site is to the east and the agriculture is to the southeast of the Oak Ridge Reservation

**Fig. 3:**
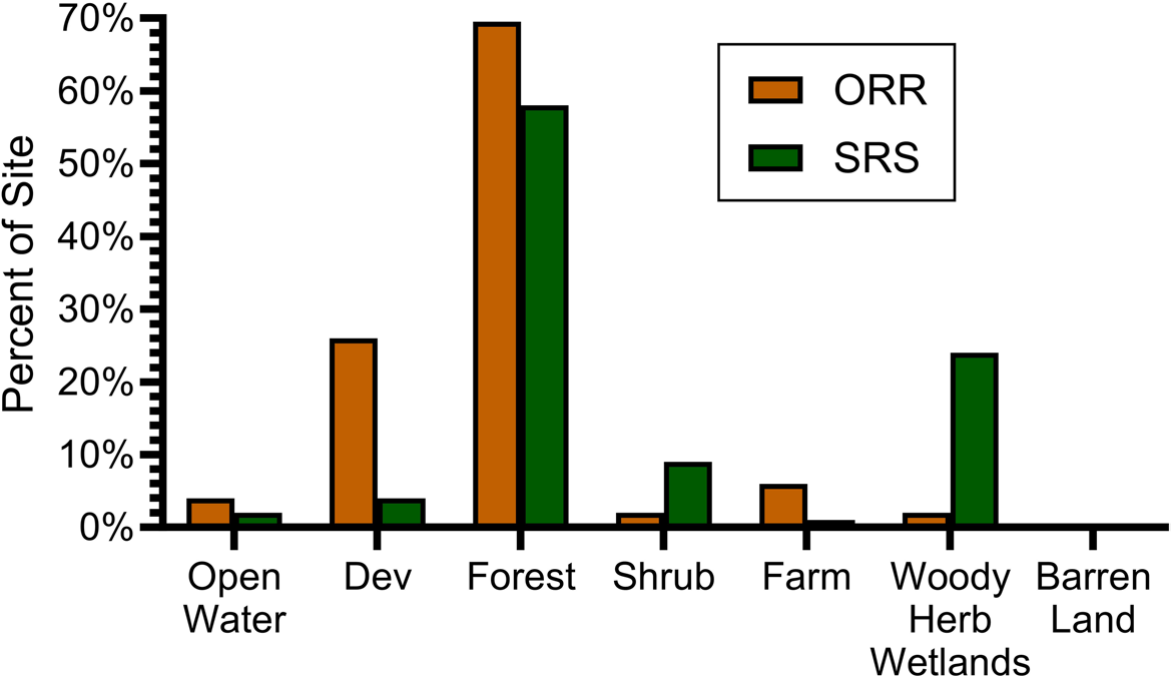
Comparison of major land use types on Oak Ridge Reservation (ORR) and Savannah River Site (SRS) (after NLCD, 2019). ORR has more forests and less woody herb wetlands than SRS

Forest accounts for 70% of ORR, with 16% developed land, and 6% in agriculture (Figs. 2 and 3). In comparison, only 45% of the 10-km buffer and 52% of the 30-km buffer are forested. Development was 30% on the 10-km buffer, and 22% on the 30-km buffer. The percentage of agriculture was also higher on the two buffers than on the site itself (Table 1).

**Fig. 4:**
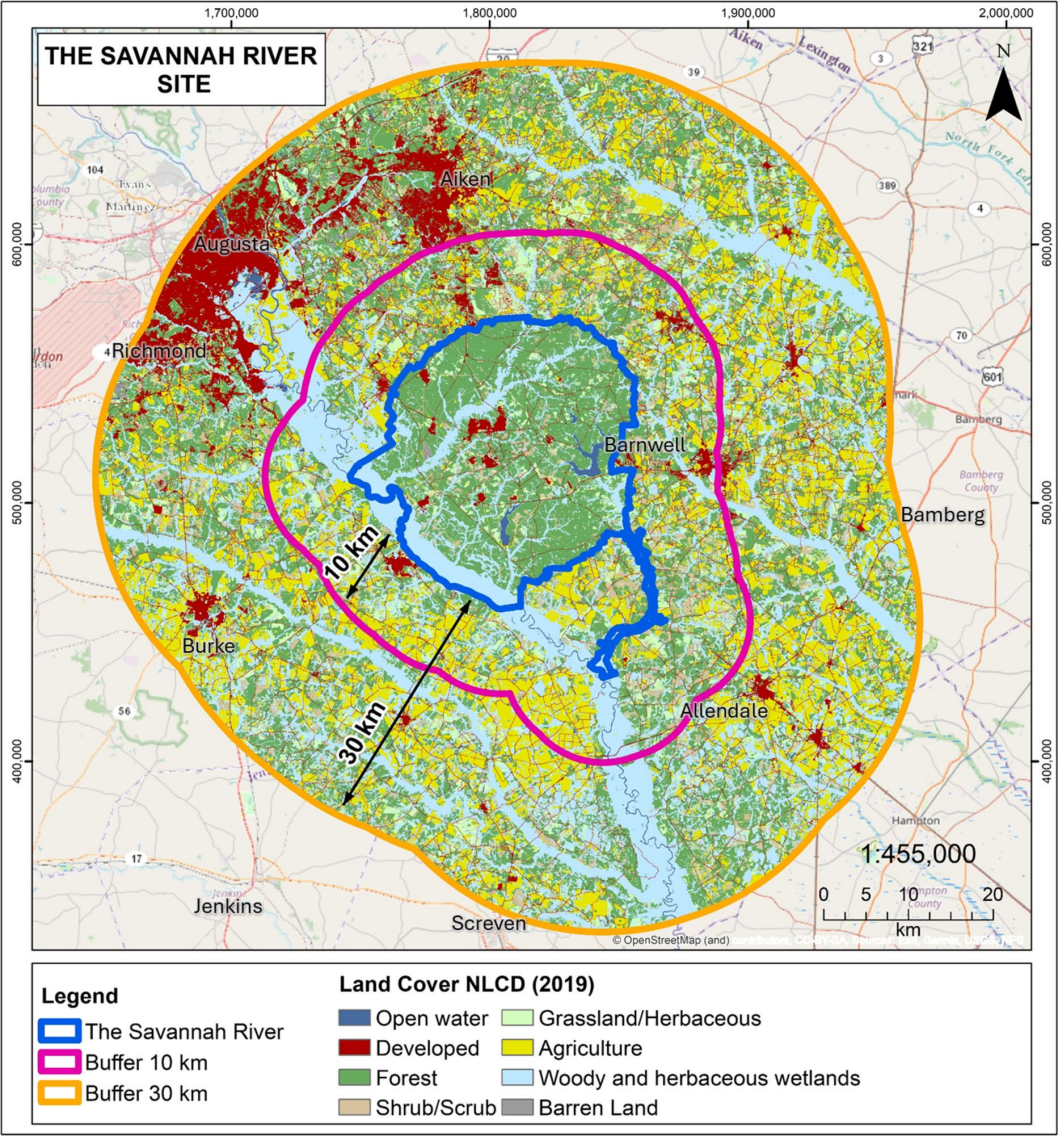
Land cover types on Savannah River Site (SRS) (bordered in blue), and the 10-km and 30-km buffer (after NLCD, 2019). Developed areas are mainly to the northwest of SRS, while agriculture is mixed throughout the buffer lands around SRS

For SRS, 58% of the land is forested, only 4% is developed, and less than 1% is in agriculture (Figs. 3 and 4). In comparison, only 28% of both the 10-km and the 30-km buffer around SRS are forested. The percentage of agriculture is less than 1% on SRS, but 20% in the two buffers around SRS (Table 1). For both sites, *X*^2^,comparing the DOE site with the 10-km buffer band and with the 30-km buffer band is significant (*P* < 0.001).

### Forest type abundance

**Fig. 5:**
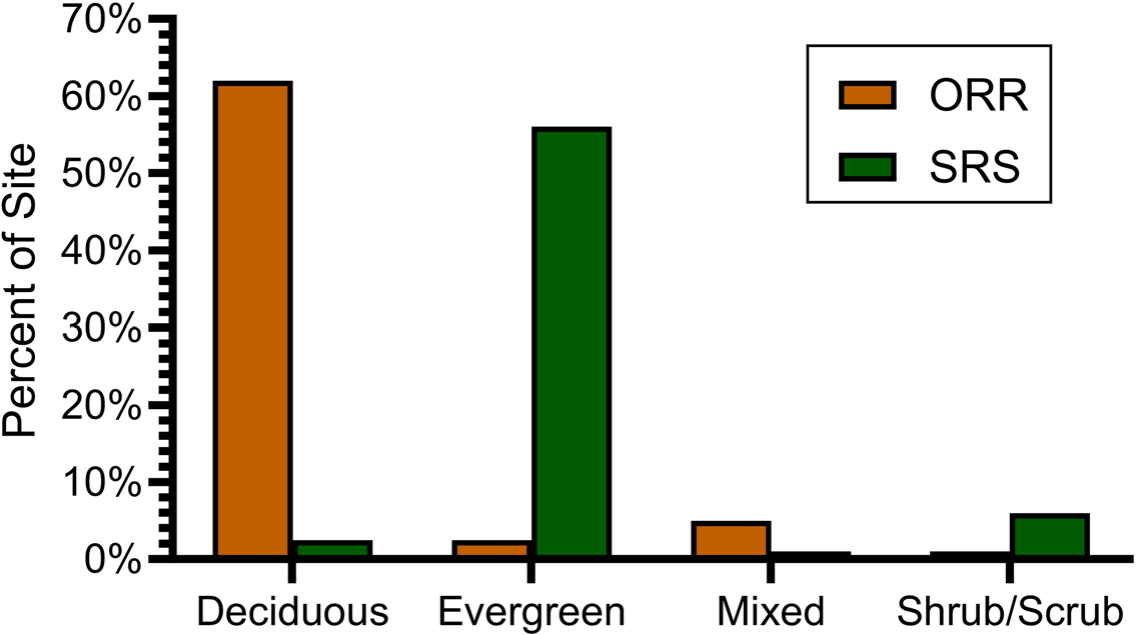
Types of forest on Oak Ridge Reservation (ORR) and Savannah River Site (SRS) from the NLCD (2019)

An examination of the type of forest from the NLCD (2019) indicates that ORR is mainly deciduous forest with a deciduous buffer; SRS is mainly coniferous with a coniferous buffer (Fig. 5). Dominant vegetation types at each site are natural for their location and topography. This is likely to indicate that any clear-cutting of forests was completed at a similar time and that on the DOE sites and local regions, some land that was once agricultural has reverted to the climax vegetation.

### Development type

There is a higher percentage of land in development on ORR compared to SRS (Fig. 3). ORR has a higher percentage of high-intensity development than does SRS. SRS has a higher percentage of development in open space than ORR (Table 2).

**Table 2:** Type of development present on Oak Ridge Reservation (ORR) and Savannah River Site (SRS) (NLCD, 2019)

DOE site	Type of development	Site		Buffer 10 km		Buffer 30 km	
		Area (ha)	%	Area (ha)	%	Area (ha)	%
Oak Ridge Reservation	Open space	541.6	25.7%	10,084.3	37.0%	39,815.8	39.2%
	Low intensity	602.5	28.5%	9781.7	35.9%	34,610.6	34.1%
	Medium intensity	520.8	24.7%	5645.9	20.7%	20,125.5	19.8%
	High intensity	446.8	21.1%	1713.8	6.3%	6963.1	6.9%
	*X*^2^ (*p*)			5.33 (0.02)		5.33 (0.02)	
Savannah River Site	Open space	1,425.1	46.5%	6554.5	58.7%	32,201.8	47.0%
	Low intensity	699.8	22.8%	3330.2	29.8%	23,876.5	34.8%
	Medium intensity	563.5	18.4%	855.9	7.7%	9059.1	13.2%
	High intensity	377.9	12.3%	428.1	3.8%	3382.3	4.9%
	*X*^2^ (*p*)			1.33 (NS)		5.33 (0.02)	

## Discussion

The methods applied here demonstrate that managers and others of degraded sites can use the NLCD (2019) as a tool to evaluate whether the proposed action will protect ecological resources on their sites, whether these resources are poorly or well represented in the region, whether they have protected more of these resources than other sites or off-site areas, and whether the climax vegetation can be used as a local indicator of habitat protection. The method also can be applied in making remediation decisions in the UK, Wales, and other countries where bioremediation or other remediation techniques are considered (Cappuyns, 2016; Latawiec et al., 2010) or in Poland where ranking of contaminated sites is necessary (Pizzol et al., 2011). It equally applies to providing information so that evidence-based decision-making can occur, for example, in managing large marine parks in Australia, South America, and elsewhere (Chiochetta et al., 2013; Cvitanovic et al., 2016). Our method involves using the amount of climax vegetation (e.g., ecoregion, EPA, 2024a) as the indicator of health within an area. It has the advantage that it is not necessary to have the exact same species or ecosystem type to allow comparison among sites; instead, the relative abundance of local climax vegetation can be compared. Below, we discuss the selection of indicators (climax vegetation), usefulness of comparing on-site and off-site climax vegetation, importance of using climax vegetation as a communication tool, and importance of being able to conduct on- and off-site assessments to use for future monitoring. For example, communication strategies for human and ecological health often involve collaborations among several nations (e.g., 17 European countries, Exley et al., 2015). It also provides information for including sustainability in remediation decisions (Braun et al., 2019).

### Selection of land use as an indictor

Evaluating ecological resources on degraded or contaminated sites allows managers to determine if ecological resources are at risk (Harris & Harper, 2000; Burger, 2008, 2019; Hou and Tabbaa, 2014; Wagner et al., 2015). There are several classes of indicators that are used for assessing the protection of ecological resources on contaminated lands, including endangered and threatened species, populations, biodiversity, and ecosystem services (NRC, 2000; Muller & Lenz, 2006; Lamb et al., 2009; Cappuyns, 2016; Siddig et al., 2016; Burger et al., 2022a, 2022b). Both DOE sites in this study have a suite of indicators that they use, including threatened and endangered species, common species whose populations might be shifting, or eco-cultural resources (Jang and Koo 2019; USDA 2020; Burger et al., 2022b). Developing indicators for the abovementioned endpoints is time-consuming and expensive and requires technical resources. Further, the same indicators cannot be used across a wide geographical area because species and vegetation types differ, and what is useful as an assessment tool in one area may not be relevant to another area.

Here we provide an alternative method that assesses the relative abundance of vegetation types of each site, rather than indicators that are generally site-specific. The method is broadly applicable because: (1) every location has a climax vegetation type (e.g., deciduous trees, grass, shrubs); (2) the natural, dominant, climax vegetation can be determined from the NLCD (2019) easily and quickly; (3) climax vegetation types within a management area can be compared with the local region; and (4) the protective value of potential management can be compared between sites, even if they do not occur within the same region. Further, the natural climax vegetation usually encompasses many of the common and rare species and ecological communities, as well as including the species that are often used as indicators of contamination (Burger & Gochfeld, 2022; Forman, 1995).

At the sites studied in this paper, the important ecological trait may well be the quality of the habitat, rather than just amount. For example, many neotropical migrants and other animals (many threatened or imperiled) require large tracts of unbroken forest for nesting (Fischer & Lindenmayer, 2007; Kroodsma, 1984). Even small industrial developments and roads through forests can degrade the habitat, often leading to increasing the number or abundance of invasive species (Larson et al., 2011; Poodat et al., 2015; Turner & Gardner, 2015). ORR and SRS both have large tracts of forest with no development, providing DOE with the opportunity to continue to protect this valuable resource regionally. At both sites, DOE has an opportunity to determine the amount and location of unbroken interior forest tracts they have and can plan to disrupt as little as possible during remediation and siting of mission-related structures. They can also maintain only narrow roads that do not open up the canopy.

### Comparing land use on- and off-site: opportunities for ecological protection

As mentioned above, DOE-EM has a large remediation task and is not expected to complete the task at some sites until the end of this century (DOE, 2022a). Their mission includes cleanup while being protective of human health and the environment. In this paper, we present a method of comparing the on- and off-site natural vegetation types, development types, and agriculture (NLCD, 2019). The method is available for any site in the U.S., uses consistent land use categories, and provides an indication of the type of development, and land cover/uses are updated every few years. This allows the sites, regulators, and the public to track changes in sites in their neighborhood.

Comparing the abundance of land use types on- and off-site allows managers to determine how much of the major natural vegetation type occurs on site and whether the parcels are large or small, fragmented or not. Time-series analysis of land-use data can identify growing threats due to development in each area. While the forest at both ORR and SRS are not imperiled, interior forest habitat is limited at ORR (Burger et al., 2023) and may be more limited in the future as roads and development lead to fragmentation (Forman, 1996; Riitters et al., 2000). Development on-site not only has the potential to destroy habitat, but to fragment nearby habitat and to increase the potential for invasive species to spread. New or improved roads on both sites increase vehicular traffic and the potential for isolating habitat fragments. The present study serves as a baseline for future evaluations.

The ecological information in this report also can inform you about the planning and execution of routine maintenance activities and future remediation so that some effects can be mitigated. Both sites have wetlands associated with forests, as determined by inspection of the wetlands within forested areas in the NLCD, and they provide unique ecological conditions, for amphibians as an example. Further, both sites have remaining remediation activities, and have a future mission. This means that they are still siting waste sites and waste management buildings, as well as the potential for siting new buildings and facilities for their continued mission. Managers can use information on forest integrity along with wetlands information to designate key ecological hotspots. In some cases, the NLCD shows wetlands that are surrounded by forest; in others, the wetlands are themselves extensive (and have their own climax vegetation).

The differences in the percentage of climax vegetation that is present on-site versus in the buffer areas for the DOE sites differ and provide a unique opportunity for planning during remediation. While both DOE sites have preserved more climax vegetation on-site than off-site, the percentage on SRS and in the region is lower than that for ORR. Thus, in some ways, the climax forest on SRS is more in need of protection simply because there is less, and the placing of any new facilities or waste sites in this vulnerable forest habitat may pose a significantly greater risk. Further, simply comparing the maps of the two sites indicates that there are large tracts of climax forest on ORR that lead into climax forest in the 10-km and 30-km buffers, whereas the large area of climax forest on SRS is not connected to any obvious large area of forest in the buffers. This provides a unique opportunity for DOE to preserve a regionally important percentage of forest on SRS, and to work with community neighbors to aid in preserving any off-site forest that currently connects with the climax forest on SRS.

### Comparisons of land use as a communication tool

DOE-EM and other governmental and industrial facilities have a responsibility to protect human health and the environment, and this responsibility extends to providing others with assurances of meeting this goal, including Congress and the public. Having tools to quickly present information to assess protection is useful both for a communication goal, but also to inform management and regulators about potential losses of key ecological resources. The natural climax community in an area is typically an important regional resource whose preservation bears watching. Management agencies must be able to communicate how proposed actions either threaten or provide opportunities to protect and improve such resources.

**Fig. 6:**
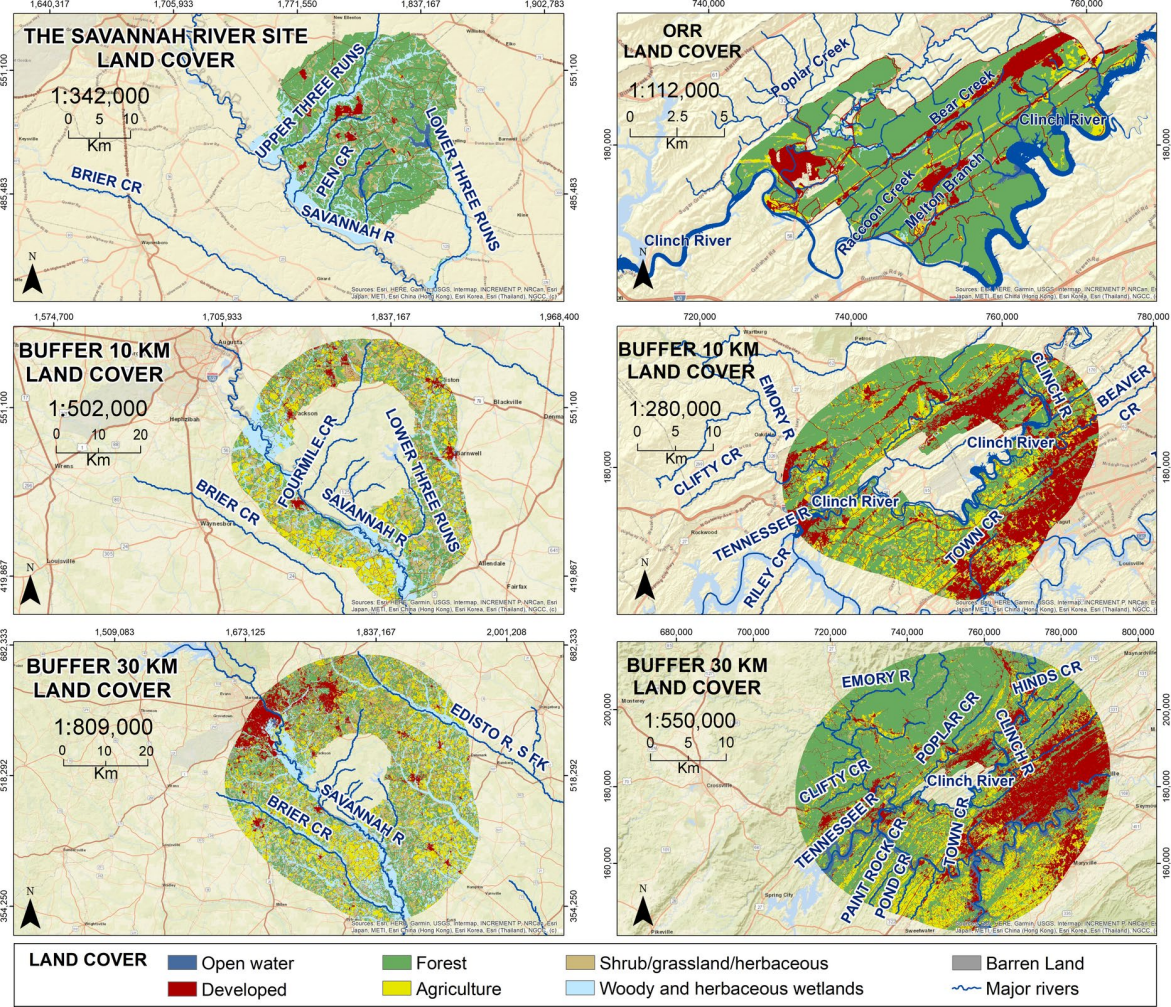
Comparison of the land cover on each site with the 10-km and 30-km buffers (from NLCD, 2019) that can be used to visually compare different sites for diverse audiences. ORR is the Oak Ridge Reservation

Visualizations provide an immediate summary of land use and habitat presence (Figs. 2 and 3). For example, comparing the sites reported in this study visually illustrates the amount of forest, and the amount of development. Further, it illustrates graphically where each land use type is located, how fragmented natural habitats are (e.g., forests), and whether there are corridors between and among forest patches, and between and among development areas. The red color of development on these maps makes the extent of development clear (Fig. 6). One important opportunity our analysis provides is the identification of valuable, intact forest patches that connect with the surrounding communities, providing green corridors. Such corridors not only serve wildlife but provide access for community members.

It is visually clear from Figs. 2 and 3 that development is closer to ORR than it is to SRS and that there is more development around ORR than around SRS. This is useful because DOE and the public can then compare land cover among different facilities. This will allow DOE headquarters and sites to consider which sites have the most important ecological resources across the complex for the purpose of devoting funds to preservation or partnering with other agencies to preserve sensitive habitat (e.g., USFWS or state resource agencies). Such prioritization is especially important if funds or personnel are limited. The technique of examining climax vegetation abundance on different DOE sites located in different parts of the country with different eco-regions allows rapid comparisons. Which DOE sites have the most critical ecological resources deserving preservation? In both sites examined, forest extends out to the community, offering an opportunity to provide direct access if communities acquire the land or develop easements, increasing access to green spaces and recreational opportunities. Further, the information can be used by community members to assess the relative importance of specific habitat types; this information is useful for community meetings with DOE at each site.

Both the actual percentages of the climax vegetation types and the visualizations (e.g., land use maps) can be used for immediate assessment and for long-term biomonitoring. Such monitoring will become even more important in the future, particularly for sites located near population centers where land use change is rapid, and opportunities for the preservation of green spaces are becoming more limited.

## Conclusions

The methods used in this paper provide a model for stakeholders using an ecological context for the condition of DOE lands and potential future management activities. The methods allow assessment of the relative abundance of climax vegetation on DOE lands compared to the region. Additionally, connectivity to the same habitat off-site can be examined. Some of the threats to the land, especially on the DOE site itself, come from routine management, surveillance, and remediation, as well as future mission-related development. These activities not only can affect the amount and quality of habitat but have the potential to introduce invasive species and subsidized predators. Understanding the value of on-site resources compared to off-site resources provides an opportunity for DOE to implement measures both during and following remediation that protect valuable ecological resources. The maps and diagrams provide a useful tool for quickly assessing the presence and potential risk to these vegetation communities for agencies and the public, allowing for both a rapid assessment, and a basis for long-term monitoring. Further, the method can be applied to DOE and other publicly held sites across the country, and to other degraded sites, whether impacted by contamination or other stressors. Because the NLCD is updated regularly, it can be used to examine temporal trends within and among regions in the U.S. as far back as the 1940 s and 1950 s. For future monitoring, the NLCD and the methods explored here can quantify changes that occur because of new industrialization, recreational use, or other on-site land conversions.

## Data Availability

The author declares that the data supporting the findings of this study are available within the article.
